# Emerging contaminant exposure to aquatic systems in the Southern African Development Community

**DOI:** 10.1002/etc.5284

**Published:** 2022-01-25

**Authors:** Kgato P. Selwe, Jessica P. R. Thorn, Alizée O. S. Desrousseaux, Caroline E. H. Dessent, J. Brett Sallach

**Affiliations:** ^1^ Department of Chemistry, University of York Heslington York UK; ^2^ Department of Environment and Geography, University of York Heslington York UK; ^3^ African Climate and Development Initiative University of Cape Town Cape Town South Africa

**Keywords:** Africa, Aquatic systems, Emerging contaminants, Environment, South African Devlopmental Community

## Abstract

The growing production and use of chemicals and the resultant increase in environmental exposure is of particular concern in developing countries where there is rapid industrialization and population growth but limited information on the occurrence of emerging contaminants. Advances in analytical techniques now allow for the monitoring of emerging contaminants at very low concentrations with the potential to cause harmful ecotoxicological effects. Therefore, we provide the first critical assessment of the current state of knowledge about chemical exposure in waters of the Southern African Developmental Community (SADC). We achieved this through a comprehensive literature review and the creation of a database of chemical monitoring data. Of the 59 articles reviewed, most (*n* = 36; 61.0%) were from South Africa, and the rest were from Botswana (*n* = 6; 10.2%), Zimbabwe (*n* = 6; 10.2%), Malawi (*n* = 3; 5.1%), Mozambique (*n* = 3; 5.1%), Zambia (*n* = 2; 3.4%), Angola (*n* = 1; 1.7%), Madagascar (*n* = 1; 1.7%), and Tanzania (*n* = 1; 1.7%). No publications were found from the remaining seven SADC countries. Emerging contaminants have only been studied in South Africa and Botswana. The antiretroviral drug ritonavir (64.52 µg/L) was detected at the highest average concentration, and ibuprofen (17 times) was detected most frequently. Despite being the primary water source in the region, groundwater was understudied (only 13 studies). High emerging contaminant concentrations in surface waters indicate the presence of secondary sources of pollution such as sewage leakage. We identify research gaps and propose actions to assess and reduce chemical pollution to enable the SADC to address the Sustainable Development Goals, particularly Goal 3.9, to reduce the deaths and illnesses from hazardous chemicals and contamination. *Environ Toxicol Chem* 2022;41:382–395. © 2022 The Authors. *Environmental Toxicology and Chemistry* published by Wiley Periodicals LLC on behalf of SETAC.

## INTRODUCTION

There is growing production and use of chemicals worldwide with estimates of more than 250,000 unique chemicals registered for use throughout the world (Wang et al., 2020). With increasing use, environmental exposure is a major concern for human and ecosystem health (United Nations Environment Programme [UNEP], 2019). Many countries and regional political unions have regulatory and policy frameworks for managing waste and chemicals, complemented by joint international actions given that pollutants undergo long‐range transport across national boundaries and are present in many countries. Because of increasing concerns, at the 2020 United Nations Environment Assembly, a group of researchers from eight countries called for a global science‐policy body to coordinate efforts to manage chemical waste in the environment (Wang et al., 2020). In Southern Africa, toxic chemical pollution is of particular concern because of a rapidly growing population and rising levels of resource consumption coupled with a lack of appropriate waste disposal technologies and methodologies, inadequate access to sanitation services, and poor enforcement, resulting in the illegal disposal of domestic and industrial waste. Similarly, investments in infrastructure, agro‐processing, pharmaceutical, automotive, energy, and mining industries are growing, but many authorities lack the diagnostic capacity to evaluate their contributions to chemical contaminant release and alignment with international standards (e.g., the Basel Convention) to ensure adequate prevention, minimization, management, and disposal of hazardous waste, considering all the social, technological, environmental, and economic aspects (Fasinu & Orisakwe, 2013).

Environmental contaminants in water have been studied for decades, initially focusing on legacy contaminants such as heavy metals and persistent organic pollutants. More recently, advances in analytical techniques have allowed for the determination of emerging contaminants (e.g., pharmaceuticals and personal care products [PCPs]) in the environment at low pg/L concentrations (Adeleye et al., 2021; Snow et al., 2020). Emerging contaminants are collectively referred to as newly synthesized and natural chemicals unintentionally released into the environment that have the potential to cause adverse ecological or human health impacts (Bellenger & Cabana, 2014; Sorensen et al., 2014). Emerging contaminants include classes of compounds (such as microplastics, nanomaterials, pharmaceuticals, PCPs, endocrine‐disruptive compounds [EDCs], steroids, flame retardants, illicit drugs, and hormones) that are not explicitly defined in environmental regulations although frequently detected in the environment (Fontes et al., 2020).

Emerging contaminants are emitted into the environment via several pathways. Chemicals that are ingested such as pharmaceuticals and illicit drugs are only partially utilized by the body and are then passed in urine and feces (Zuccato et al., 2006). Others, such as PCPs or cleaning products, are washed down the drain (Daughton & Ternes, 1999). Where infrastructure exists, these compounds are conveyed through sewage systems to wastewater treatment plants (WWTPs; Chiavola et al., 2019). Conventional WWTPs are not typically designed for the removal of these compounds, and therefore many chemicals are inefficiently removed via the treatment process. For example, Gani et al. (2021) revealed that the removal efficiencies of antiretrovirals and polybrominated diphenyl ethers in South African treatment plants varied greatly from no removal to complete removal. Those emerging contaminants that are not removed are emitted into the rivers, streams, and coastal waters that receive treated wastewater effluent (Deblonde et al., 2011). Alternatively, combined wastewater and stormwater systems, typical of aging infrastructure, result in routine emissions of untreated sewage during high flow events following heavy rainfall (Devault et al., 2018). Contaminants such as pesticides and hormones are also transported into water sources via surface runoff that eventually reaches rivers and is then ultimately transported into estuaries and aquifers (Pal et al., 2010).

Although many of these emerging contaminants are persistent and can be found at high concentrations in the environment, most are subject to degradation and transportation processes in the environment. However, emerging contaminants can exhibit pseudo‐persistent behavior because they are continuously released into the environment, mainly through WWTP effluent, and thus maintain relatively stable levels in water bodies receiving effluents (Fontes et al., 2020; Mhuka et al., 2020). This is because the rate of replacement of these compounds in the environment can be higher than the rate of their removal or degradation (Brain et al., 2008). Because many of these compounds are biologically active, because they are designed to affect biological processes at low concentrations, and because many wastewater treatment technologies are inefficient at removing such compounds, it is critical that we monitor their occurrence in the environment (Ankley et al., 2007) and use this information to mitigate impacts. The potential impacts of emerging contaminant pollution are widespread, and the cost is not typically born by the polluters, but by other, often marginalized, uninformed communities living adjacent to water bodies and depending on water sources for livelihoods (e.g., subsistence and commercial farming, pastoralism, fishing, drinking, washing).

The ecotoxicological effects of emerging contaminants can be harmful. For example, 17‐α‐ethinylestradiol, a synthetic estrogen used in birth control pills, was found to lead to feminization of male fish after chronic exposure at concentrations of 5–6 ng/L (Kidd et al., 2007). Although emerging contaminants have been detected in aquatic organisms within the Southern African Development Community (SADC; Chokwe et al., 2015; Groffen et al., 2018; Rimayi et al., 2018), investigations into their effects in the region are still lacking. Similarly, the continuous release of antibiotics has been linked to the increase in antibiotic‐resistant genes and bacteria, which ultimately compromises the efficiency of antibiotics (Murray et al., 2021; Rizzo et al., 2013). This affects both human and aquatic life because it contributes to the drug resistance in pathogens that cause serious diseases such as malaria and cholera (Fekadu et al., 2019). Similarly, the emergence of antibiotic‐resistant bacteria threatens soil and faunal microbiomes, thereby hindering ecosystem services such as nutrient cycling, pollutant remediation, and synthesis of bioactive compounds such as antimicrobials (Gwenzi & Munondo, 2008; Williams‐Nguyen et al., 2016; Zhu et al., 2019). Furthermore, the bioaccumulation of highly persistent per‐ and polyfluoroalkyl substances (PFAS) combined with their potential to act as EDCs has increased concern considering their ubiquitous occurrence in the environment (Fauconier et al., 2020; Ssebugere et al., 2020).

Although there has been a significant increase in the assessment of emerging contaminants in the environment, most publications have focused on specific chemical groups and are biased toward developed countries. For example, in a global review of pharmaceuticals in the environment (aus der Beek et al., 2016), 221 studies were from Germany and 83 from Spain, but only 23 were from the entire African continent. Local and regional analyses are needed given the occurrence of emerging contaminants and the fact that drivers of pollution are not the same across different regions. This is particularly true for sub‐Saharan Africa because it is the second largest continent, with 70.7% of the population experiencing water stress (Macdonald et al., 2012; SDG Indicators, 2020), high levels of industrialization, urbanization, population and economic growth (Fekadu et al., 2019), and more than 75% of the population being dependent on groundwater for various industrial, agricultural, and mining activities (McGill et al., 2019; Sorensen et al., 2014). Also, integrated water management is particularly lacking (Mogomotsi et al., 2018; Rahm et al., 2006; SDG Indicators, 2020). Seventy percent of the urban population is unconnected to reticulated sewerage systems, the infrastructure is aging, and a low proportion of this population is using safely managed drinking water services (Fayiga et al., 2018). In addition, 80% of wastewater is directly discharged untreated into surface waters or soil (Ellis, 2006; Nyenje et al., 2010; Verlicchi et al., 2010; Watkinson et al., 2008), which is a growing concern for governments in terms of the import of toxic wastes such as polychlorinated biphenyls (PCBs; Breivik et al., 2011). Concomitantly, only 15% of the SADC is on track to meet the sanitation target of the Sustainable Development Goals (SDGs). The impacts of the COVID‐19 pandemic are also making the need for water, sanitation, and hygiene facilities ever more critical.

Despite the existence of frameworks, policies, institutions, and management instruments to mitigate the impacts of contaminants, and the urgent need to counter these impacts, sub‐Saharan African countries have contributed few data on the chemical contamination of aquatic systems (K'oreje et al., 2016), and only one study, in South Africa, has critically reviewed the state and occurrence of emerging contaminants (Gani et al., 2021). No pertinent data or analysis have been produced that include other countries in the SADC region.

## MATERIALS AND METHODS

We searched for peer‐reviewed articles in bibliographic databases such as Web of Science, Scopus, Science Direct, and Google Scholar, using keyword search strings including “emerging contaminants,” “water contaminants,” “wastewater,” and “SADC” along with the names of all 16 member states of SADC. Searches had no time limitation and were only in English due to the linguistic capacity of the authors. The retrieved articles were then vetted to include only the articles that reported primary data on chemical detections from environmental waters. This yielded 59 relevant articles published from January 1995 to December 2020. Data from these studies were then added to a database reporting sampling dates, year of publication, location, analytical technique, compounds monitored, and concentrations. For consistency, all concentration measurements were converted to units of µg/L. Similarly, analytical methodologies were generalized based on their underlying technology. For example, high‐performance liquid chromatography (HPLC), ultra‐fast liquid chromatography, and ultra–high‐performance liquid chromatography coupled to a mass spectrometer (MS) were generalized as liquid chromatography–mass spectrometry (LC–MS) techniques. Only those compounds above their respective limit of detection were recorded, and each one was assigned a unique Chemical Abstract Service (CAS) registry number to unify compounds with more than one name.

Water samples were classified as either wastewater, surface water, or groundwater. Groundwater was made up of samples taken from boreholes and wells. Surface water included samples from rivers, lakes, springs, dams, and deltas. Although not technically an environmental water source, we included wastewater effluent detections in the database because wastewater is known to dominate water flows during low flow events of receiving rivers in the arid regions typical of the SADC (Gani et al., 2021).

Chemicals were classified into one of eight categories: EDCs, hormones, inorganic ions, PFAS, PCPs, pesticides, pharmaceuticals, and polyaromatic hydrocarbons (PAHs). Further analysis was conducted on EDCs, hormones, PFAS, PCPs, and pharmaceuticals because they were defined as emerging contaminants. Some compounds reported fell under multiple chemical categories. In such cases, the categorization made by the respective author was given preference. All chemicals and their assigned categories can be found in the open source database. The database was built in Microsoft Excel Ver 2105 and is openly accessible https://pure.york.ac.uk/portal/files/73799193/Review_Database.xlsx, https://creativecommons.org/licenses/by/4.0/ (see the Data Accessibility Statement). Analysis was performed and figures generated in Tableau™ (Salesforce).

## RESULTS AND DISCUSSION

### Trends in monitoring studies

**Figure 1:**
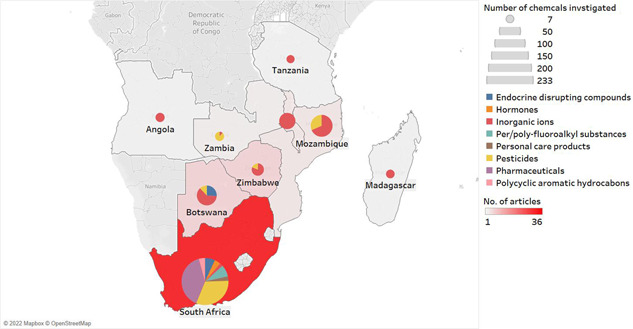
Distribution of all studies published between 1995 and 2020 on chemical pollution in aquatic systems in the Southern African Developmental Community region. The diameter of the pie chart (gray scale, top right) represents the number of unique chemicals investigated. The colors within the chart represent the chemical contaminant categories. The intensity of the shading of the country indicates the number of publications from that country, ranging from 1 to 36.

Bibliographic data indicated a recent increase in the number of studies on water contamination in Southern Africa, suggesting that calls for a more global approach to pollution are being addressed. Although the present study reviews investigations from as early as 1995, 34 of the 59 reviewed studies were carried out between 2017 and 2020. Importantly, publications from only 9 of the 16 SADC member states were found. No literature from the remaining seven countries (including Comoros, Democratic Republic of Congo, eSwatini, Lesotho, Mauritius, Namibia, and Seychelles) were found, highlighting a key knowledge gap. Furthermore, of the 59 publications meeting the inclusion criteria, 61% (36 studies) were from South Africa, as shown by the red color in Figure 1. The remaining 39% (23 studies) were conducted in the other eight countries. This may be attributed to more research funding and capacity, more environmental reporting from public agencies, or the presence of many more global technological companies in South Africa compared with neighboring countries. Alternatively, this could be due to the environmental importance of the country's highlands for water security in the Drakensberg Basin, and the growing industrial threats described in the *Introduction*.

The analysis of water samples in most of these studies was carried out with (or with the assistance of) collaborators in high‐income countries such as the United States (Bartsch et al., 2019; Mladenov et al., 2014), Switzerland (Curchod et al., 2020), and Italy (Ricolfi et al., 2020), highlighting the importance of international collaborations as well as a general lack of environmental analytical laboratories in the SADC region (K'oreje et al., 2012). South Africa is the exception, as evidenced by the emergence of locally produced data (Clement et al., 2019; Farounbi, 2020; Wanda et al., 2017). Most countries have focused on inorganic ions (Figure 1) such as sulfur, copper, and iron—again with the exception of South Africa. This may be because mining activities in Southern Africa are a traditional source of surface water pollution and so effort has been directed toward understanding their impact on the environment (Ahoulé et al., 2015). Calcium, sodium, potassium, chlorine, and sulfates are also extensively studied ions because they are routinely investigated during water quality checks of underground sources of drinking and irrigation water (Barbieri et al., 2019).

**Figure 2:**
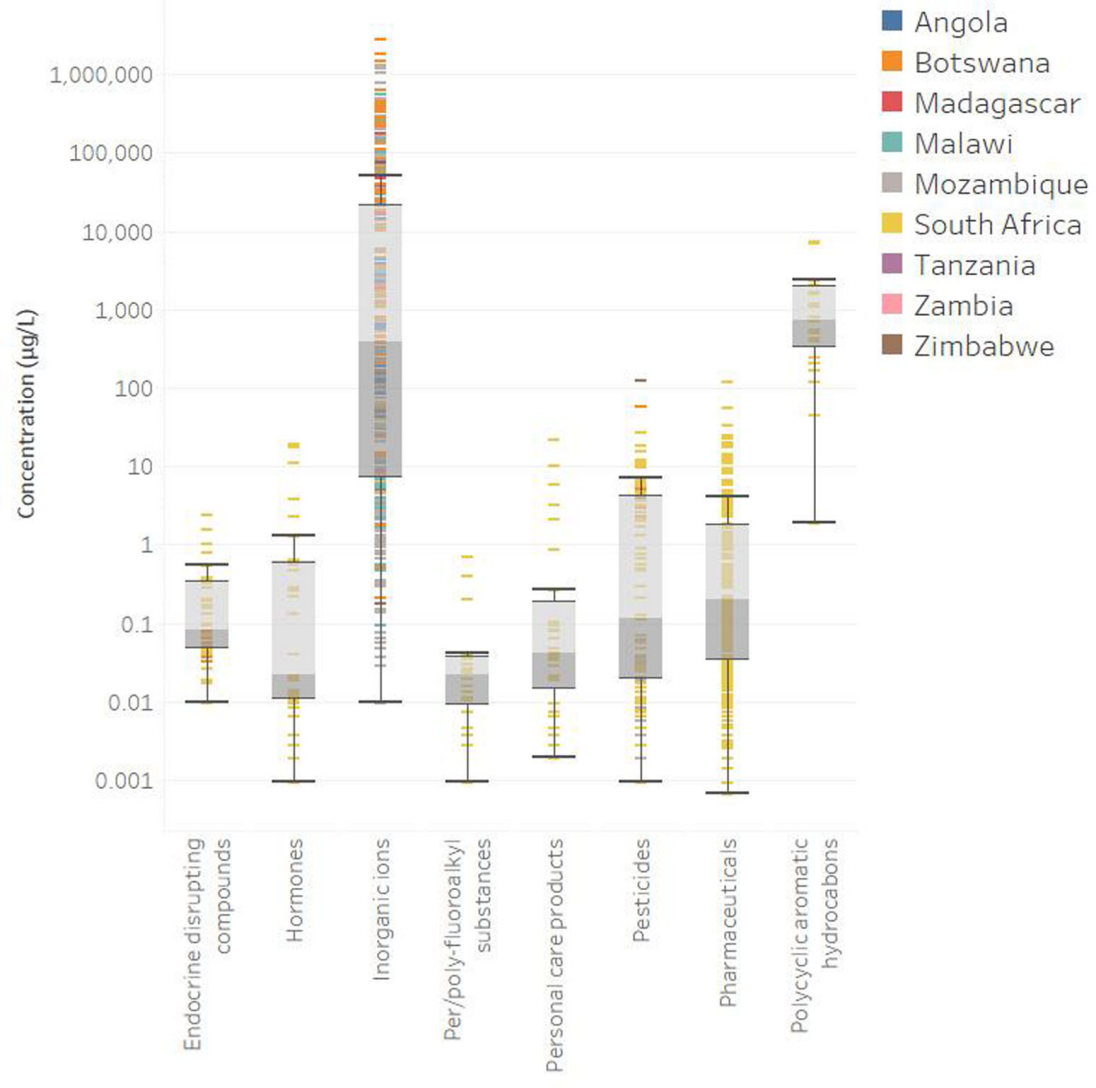
Individual detections of contaminants in aquatic systems of the Southern African Developmental Community grouped by chemical classification. Box and whisker plots show minimum, maximum, median, lower interquartile, and higher interquartile concentrations. The color of each detection represents the country of origin of the sample.

The number of chemicals investigated in each country varied greatly (Figure 1), from only 7 in Tanzania (Mdegela et al., 2009), to 233 in South Africa. We collected a total of 867 individual detections (Figure 2). Inorganic ions were the most investigated category of chemical contaminants, with the widest interquartile range of concentrations (7–24,745 µg/L) in all nine countries for which data was reported (Ricolfi et al., 2020; Schwartz & Kgomanyane, 2008). The average concentrations were 0.32, 1.28, 75,134.67, 0.07, 1.30, 2.9, 2.67, and 19 799.79 µg/L for EDCs, hormones, inorganic ions, PFAS, PCPs, pesticides, pharmaceuticals, and PAHs, respectively. South Africa was the only country where investigations of all eight categories of chemical contaminants were conducted. This finding also suggests that even where investigations of chemical pollution in water have occurred within the SADC, the scope of the chemicals monitored has been limited and for most of the SADC member states, the extent of chemical pollution cannot be properly assessed.

### Emerging contaminants in the SADC region

In recent years, scientific focus has shifted from studying legacy water contaminants (compounds generated during industrialization prior to environmental protection regulation such as pesticides and dioxins) to the study of compounds such as pharmaceuticals and PCPs (Wilkinson et al., 2017). Importantly, across the region, only two countries have produced literature on emerging contaminants, Botswana and South Africa. A total of 226 compounds were identified within five classes: EDCs, PFAS, PCPs, pharmaceuticals, and hormones. Six EDCs were detected in surface water in Botswana (Bartsch et al., 2019), whereas the remaining 220 compounds (35 EDCs, 29 hormones, 20 PFAS, 28 PCPs, and 163 pharmaceuticals) were detected in groundwater, surface water, and wastewater in South Africa.

**Figure 3:**
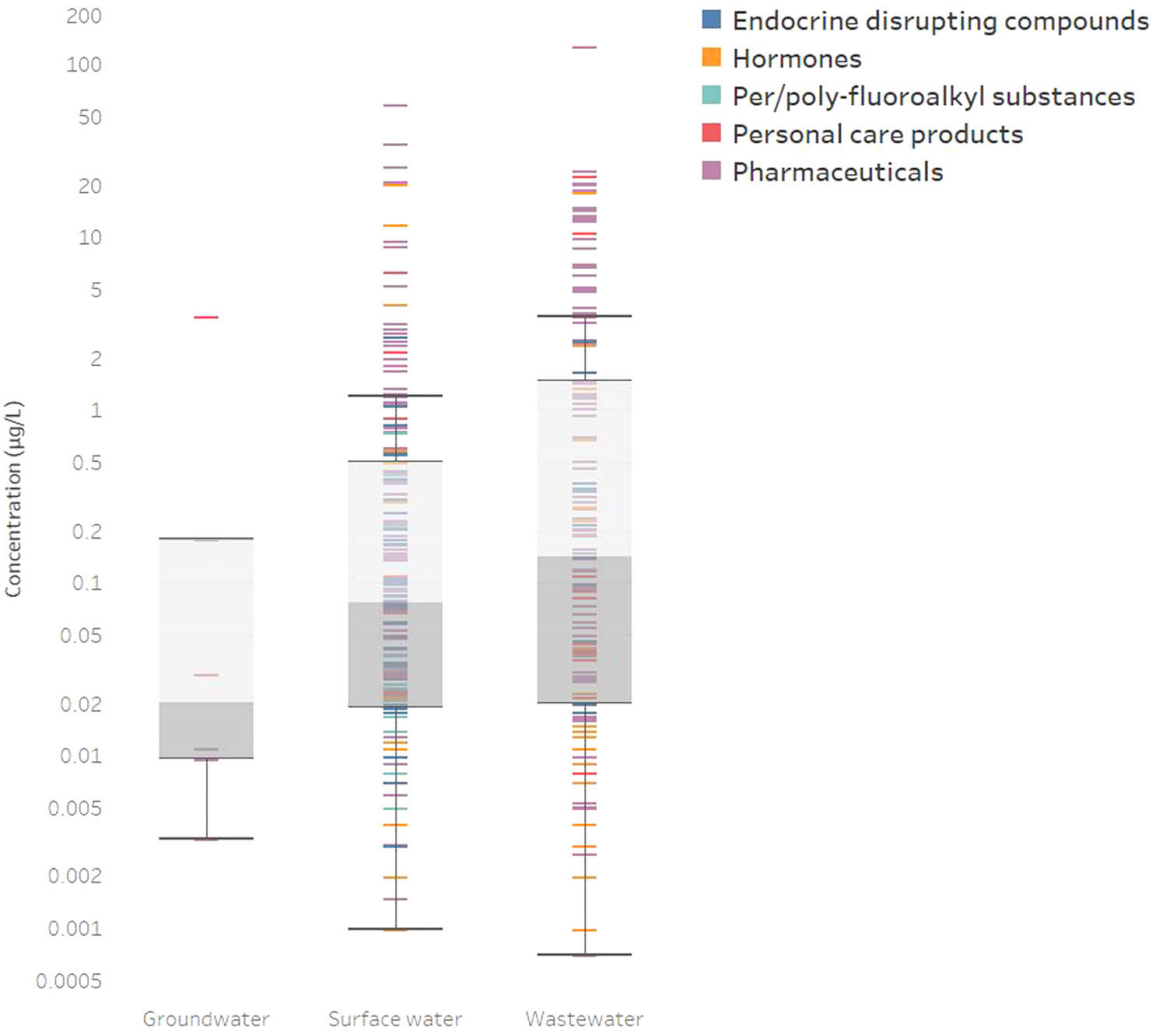
Range of emerging contaminant concentrations in groundwater, surface water, and wastewater in South Africa and Botswana, the only countries in the Southern African Developmental Community where emerging contaminants have been investigated. Colors indicate class of emerging contaminant for each detection.

The vast majority of studies, and subsequent chemical detections, were performed in surface waters (212 detections from 21 studies) and wastewater (164 detections from 12 studies), as shown in Figure 3. Only two studies investigated emerging contaminants in groundwater, which resulted in the detection of six emerging contaminants from groundwater in South Africa including bisphenol A, carbamazepine, cyclopenta, efavirenz, nevirapine, and tonalide at average concentrations of 0.18, 0.0095, 3.48, 0.0033, 0.011, and 0.03 µg/L, respectively (Rimayi et al., 2018; Wanda et al., 2017). This finding highlights the fact that groundwater remains understudied in the SADC region. The presence of emerging contaminants is concerning given the high dependence on groundwater as a source of drinking water for most of the population in this area. Further study is required to establish sources of contamination as well as the effects on human health and aquatic systems associated with continued exposure of these compounds, concomitantly with other classes of chemical pollution.

In terms of emerging contaminants, both surface water (22 studies) and wastewater (13 studies) were studied more extensively than groundwater (2 studies). The increasing number of recent publications on wastewater concentrations may be associated with a growing interest in wastewater removal efficiencies of the various treatment facilities available. A total of five studies investigated both influent and effluent flows of WWTPs—all in South Africa (Farounbi, 2020; Manickum & John, 2014; Matongo et al., 2015b; Mhuka et al., 2020; Wanda et al., 2017). Importantly, the combined data set revealed that the ranges of wastewater and surface water concentrations were very similar. This is unexpected, because surface waters typically have lower concentrations due to dilution of contaminants in baseline flows. This could be an indication that there are likely secondary sources of contamination of river waters, such as the sewage leakage and inappropriate waste management typical in municipalities and settlements with limited centralized wastewater treatment infrastructure.

For some compounds, the presence of higher concentrations downstream of wastewater treatment outputs highlights the contribution of wastewater and the inefficient removal of emerging contaminants. For example, Matongo et al. (2015a) found that the concentrations of sulfamethazine, ibuprofen, and clozapine increased in a WWTP from below the limit of detection to 1.10, 5.76–12.94, and 8.95–14.43 µg/L, respectively. This was believed to be due to chemical reactions during the treatment process that convert unmeasured human metabolites and/or transformation products back to their original parent compounds. In another study, the same group found that clozapine was not detected in the WWTP influent water, but was detected in the effluent at concentrations up to 9.56 µg/L (Matongo et al., 2015b). Mhuka et al. (2020) also observed this phenomenon, suggesting that the detection of compounds in the effluent waters was caused by a possible storage of these compounds within the treatment plant.

**Figure 4:**
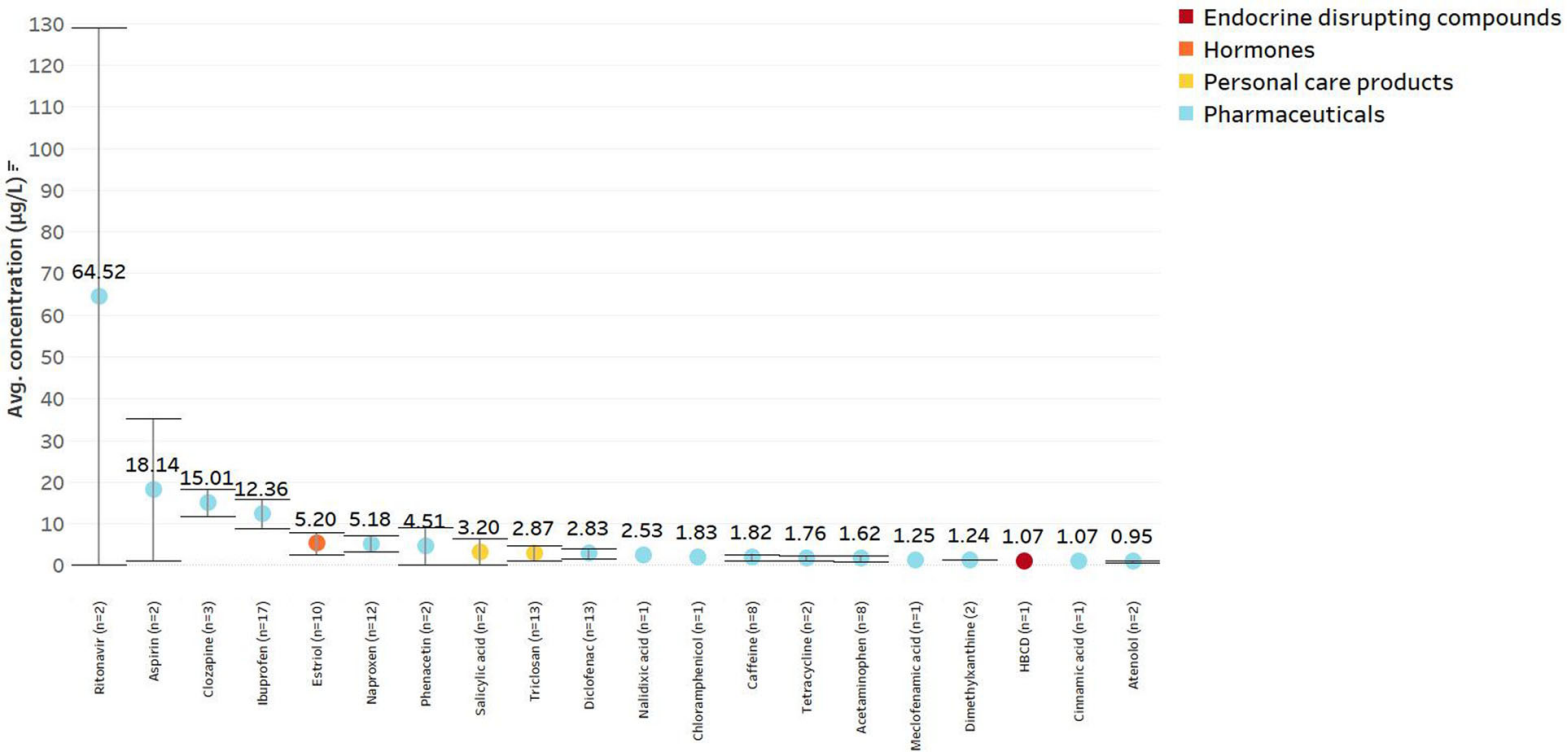
The 20 emerging contaminants with the highest average concentrations. Color reflects the EC category.

The emerging contaminant with the highest average concentration was ritonavir (64.52 µg/L; Figure 4), a drug used for the treatment and prevention of human immunodeficiency virus (HIV) infections (Deeks, 2018). This is indicative of the high prevalence of HIV/acquired immunodeficiency syndrome in Southern Africa (Kuteesa et al., 2019), along with secondary uses of the other antiretroviral medication (e.g., efavirenz, nevirapine) for recreational use (Grelotti et al., 2014). The second highest average concentration was for aspirin (18.14 µg/L), the most consumed pharmaceutical globally (Agunbiade & Moodley, 2016). The antipsychotic drug clozapine had the third highest average concentration, at 15.01 µg/L, followed by the nonsteroidal anti‐inflammatory drug ibuprofen, at 12.36 µg/L. Only one EDC, hexabromocyclododecane, one hormone (estriol), and two PCPs (salicylic acid and triclosan) were found in the highest 20 average concentrations (1.07, 5.20, 3.20, and 2.87 µg/L, respectively). The remaining 16 compounds were pharmaceuticals including the antibiotic compound tetracycline, with an average concentration of 1.76 µg/L. Notably, no PFAS compounds were found in the 20 highest average concentrations of emerging contaminants in the SADC.

**Figure 5:**
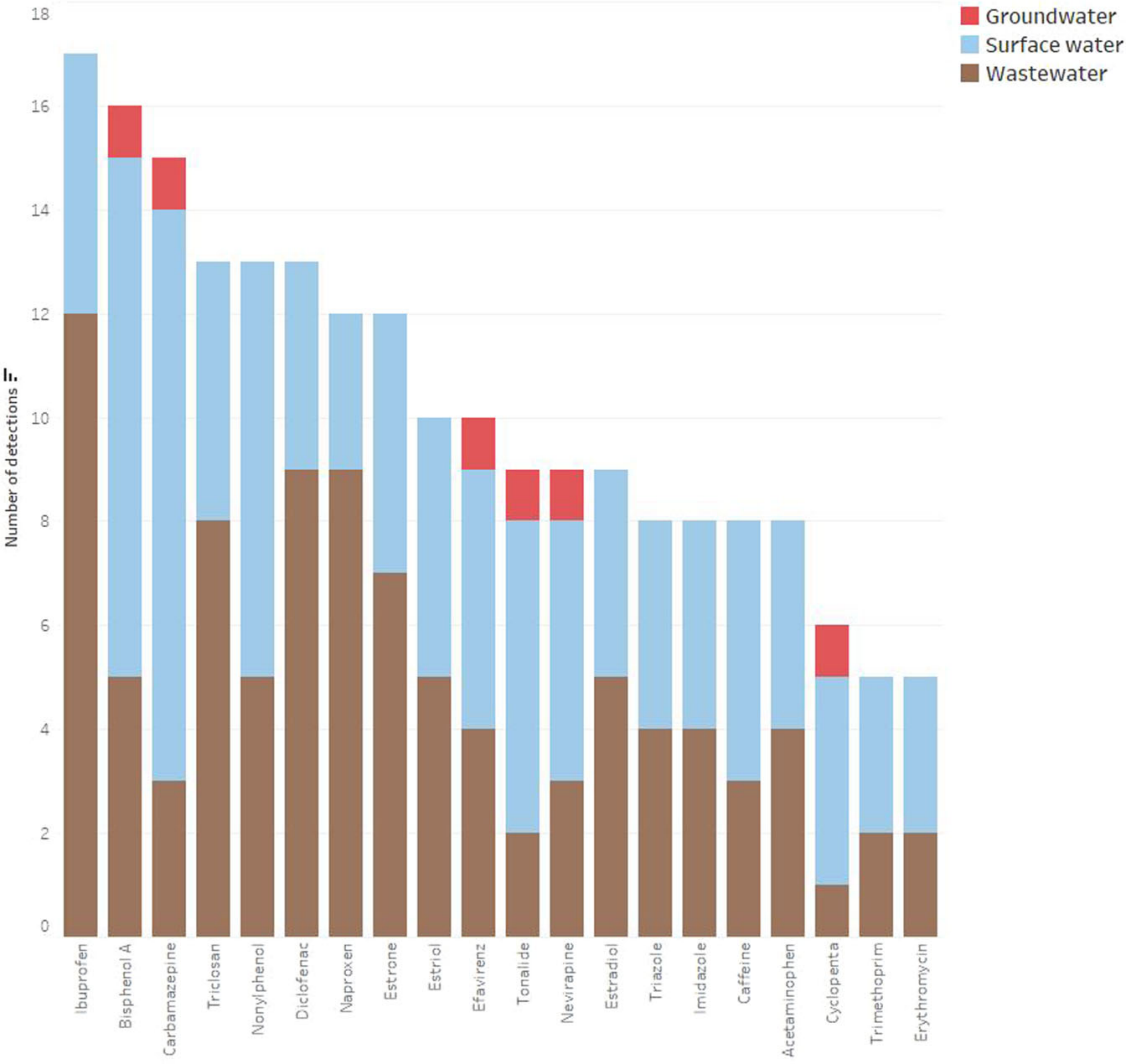
Most frequently detected emerging contaminants in wastewater (brown), surface water (light blue), and groundwater (red).

Examining the frequency of detections helps to reveal the exposure of various compounds in environmental media. A total of 20 emerging contaminants have been detected five or more times in the studies in the SADC region (Figure 5). Each of these 20 compounds was detected at least once in wastewater and three or more times in surface waters. Ibuprofen was detected the most (17 times in total, 12 times in wastewater and 5 times in surface water), followed by bisphenol A (16 times in total, 5 times in wastewater, 10 times in surface water, and once in groundwater) and carbamazepine (15 times in total, 3 times in wastewater, 11 times in surface water, and once in groundwater). Six compounds (bisphenol A, carbamazepine, efavirenz, tonalide, nevirapine, and cyclopenta) were each detected once in groundwater. Detections in groundwater are indicative of compounds that are both persistent and mobile, whereas frequent surface water detections can indicate pseudo‐persistent behavior. The fact that the 20 most frequently detected compounds were found in both wastewater and surface water suggests that wastewater treatment facilities represent an important source of emerging contaminant exposure. Two antibiotic compounds, erythromycin and trimethoprim, were among the most commonly detected compounds and were both detected twice in wastewater and three times in surface water (Table 1).

**Table 1:** Summary of the 20 most detected compounds and their categories, along with quantitative data on the number of times detected, their average concentrations, and concentration ranges

Compound	Category	No. of detections	No. of studies	Average concentration (µg/L)	Concentration range (µg/L)
Ibuprofen	Pharmaceutical	17	8	12.36	0.001–58.71
Bisphenol A	EDC	16	4	0.18	0.01–1.68
Carbamazepine	Pharmaceutical	15	6	0.23	0.0095–1.46
Triclosan	PCP	13	5	2.87	0.003–22.90
Nonylphenol	EDC	13	4	0.35	0.01–2.56
Diclofenac	Pharmaceutical	13	6	2.83	0.001–15.00
Naproxen	Pharmaceutical	12	5	5.18	0.001–20.40
Estrone	Hormone	12	4	0.01	0.001–0.02
Estriol	Hormone	10	5	5.20	0.001–20.30
Efavirenz	Pharmaceutical	10	5	0.89	0.0033–7.10
Tonalide	PCP	9	3	0.04	0.002–0.10
Nevirapine	Pharmaceutical	9	5	0.06	0.0007–0.35
Estradiol	Hormone	9	5	0.79	0.003–4.11
Triazole	Pharmaceutical	8	1	0.03	0.004–0.09
Imidazole	Pharmaceutical	8	1	0.10	0.012–0.21
Caffeine	Pharmaceutical	8	5	1.82	0.01–6.87
Acetaminophen	Pharmaceutical	8	5	1.62	0.031–6.10
Cyclopenta	PCP	6	1	0.60	0.010–3.48
Trimethroprim	Pharmaceutical	5	3	0.08	0.016–0.17
Erythromycin	Pharmaceutical	5	3	0.09	0.002–0.24

EDC, endocrine‐disrupting compound; PCP, personal care product.

### Analytical and sampling methods

Emerging contaminants have become an increasing concern largely because these compounds are likely to occur in environmental water compartments at such low levels (ng/L–µg/L) that they are a challenge to detect using existing analytical methods. Therefore, the analytical techniques employed impact the types of compounds and concentrations measured.

**Figure 6:**
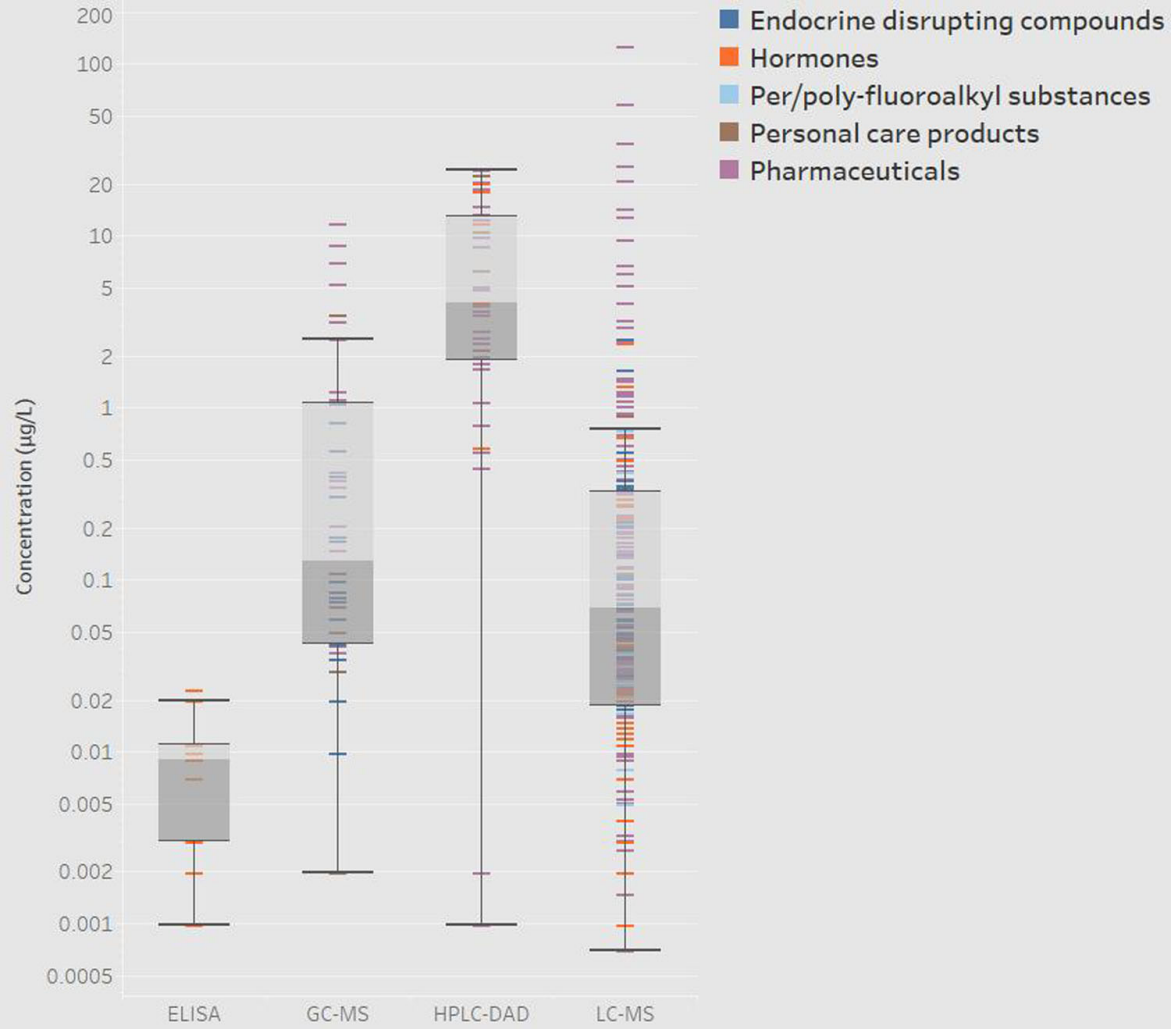
Analytical techniques used for the analysis of emerging contaminants in Southern African Developmental Community. Colors indicate class of emerging contaminant for each detection. ELISA = enzyme‐linked immunosorbent assay; GC–MS = gas chromatography–mass spectrometry; HPLC–DAD = high‐performance liquid chromatography–diode array detector; LC–MS = liquid chromatography–mass spectrometry.

Most emerging contaminants were detected with LC–MS instrumentation (266 times in total: 166 pharmaceuticals, 18 PCPs, 25 PFAS, 31 hormones, and 26 EDCs), followed by GC–MS (64 times in total: 26 pharmaceuticals, 15 PCPs, and 23 EDCs), and HPLC–diode array detector (DAD; 40 times in total: 31 pharmaceuticals, 4 PCPs, and 5 hormones), as shown in Figure 6. Enzyme‐linked immunosorbent assay (ELISA) kits resulted in the lowest number of individual detections (five detections, all for hormones). The ELISA kits provide a rapid, often inexpensive yet reliable method for screening and quantifying biological matrices such as proteins (Horn et al., 2019) in clinical, forensic, and environmental testing facilities (Barnes et al., 2015). Thus the technique has been limited to detecting hormones (Cornelius et al., 2011) in the SADC region. However, ELISA does not require expensive equipment or a high level of user expertise. As a technique, it does have documented issues with selectivity and sensitivity and is therefore normally used alongside techniques such as LC–MS in environmental monitoring (Aga et al., 2005).

The HPLC–DAD method was used to detect both hormones and pharmaceutical compounds. However, this technique only detects compounds occurring at relatively high concentrations or with significant sample preconcentration, which is evidenced by the high average concentrations reported with this technique (Figure 4). The HPLC–DAD technique lacks the selectivity of HPLC coupled with MS but is cheaper in terms of both capital investment and operational costs. On the other hand, both GC and LC techniques coupled with MS allow for the measurement of a wide range of compounds with a large range of concentrations.

The LC–MS method was used more frequently than GC–MS to detect and quantify emerging contaminants, because it is the preferred method for the analysis of polar, nonvolatile compounds, whereas GC techniques are limited to volatile compounds (Gumbi et al., 2017) or require derivatization during sample preparation. Derivatization is the process by which a chemical compound is converted to a similar product that has desired properties such volatility, solubility, stability, and detectability (González‐Mariño et al., 2010). For example, derivatization techniques are commonly used for the analysis of the glucuronic and sulfuric metabolites of hormones by GC–MS (Mnguni et al., 2018).

Sample collection and storage of environmental water samples play important roles in achieving quality results. In the studies conducted in the SADC region, a range of methods was used to sample and store water—often based on the availability of sampling kits, the matrix being sampled, the sampling volume collected, and the environmental risks (e.g., presence of dangerous wildlife; Alimi et al., 2021). Approximately 90% of the investigations reviewed employed grab sampling as a primary method of collection. In most cases, samples were stored in amber glass vials, protected from UV radiation, and stored at 4 °C or in cooler boxes during transportation (Kanama et al., 2018; Mnguni et al., 2018; Sturve et al., 2016). In a few studies, polypropylene bottles were used as sample storage vessels (Fagbayigbo et al., 2018). Other sampling strategies included passive sampling using Chemcatchers® (polystyrene divinylbenzene–reverse phase sulfonated) cartridges (Curchod et al., 2020). Passive samplers provide the opportunity to measure low concentrations over a longer time course (7 days or more) and are used to determine time‐weighted average concentrations. Very few studies (approximately 20%) clearly indicated exact locations of sampling via global positioning system (GPS) coordinates, nor did they indicate parameters such as depth of sampling, temperature, pH, conductivity, and oxygen saturation. Storage of samples also varied greatly; some samples were filtered or were pH adjusted before storage, whereas others were stored as is. Although some samples were stored frozen, most were refrigerated at 4 °C until analysis.

The design of monitoring campaigns, for example timing and frequency of sampling events, plays a critical role in providing a temporal understanding of emerging contaminant exposure. The duration of sampling campaigns and frequency of sampling events varied significantly in the SADC studies. More than 80% of the studies collected samples at least twice—usually one collection during the summer months and another during the winter months, to represent the rainy season and the dry season, respectively (Selebatso et al., 2018). Pesticides in river water were subject to temporal variation between the seasons, with more detections in the summer when spraying events are common and fewer detections during the dry winter months when there are fewer spraying events (Curchod et al., 2020; Horn et al., 2019). On the other hand, the concentrations of certain hormones were found to decrease with increasing rainfall due to dilution (Manickum & John, 2014). Detections in wastewater (Archer et al., 2020) and groundwater (Selebatso et al., 2018) showed no seasonal variations. Approximately 20% of the studies conducted longitudinal sampling that took place over 12 months (Berg et al., 1995; Wooding et al., 2017) or longer (Gumbi et al., 2017; Manickum & John, 2014; Mnguni et al., 2018).

### Research gaps and recommendations

The most significant research gap identified is the lack of information regarding the occurrence of emerging contaminants, and chemical contaminants more generally in the SADC. Sixty‐one percent (39 studies) of the publications on chemical exposure in the SADC originated from South Africa alone. Similarly, of the 28 articles that measured emerging contaminants, 93% (26 studies) were from South Africa, whereas the remaining two studies (7%) were from Botswana (Bartsch et al., 2019; Mmualefe et al., 2009). Although this could be because South Africa has the highest gross domestic product in Africa, after Nigeria (Valipour, 2015), demographic, socio‐economic, and geographical drivers of pollution may also influence the availability of research outputs. As previously discussed in the *Trends in monitoring studies* section, collaborations with developed countries have been helpful in generating environmental data. Establishing regional collaborations within the SADC could improve the regional data output for environmental monitoring campaigns that would benefit many parts of the entire region because many of the rivers and streams cross international boundaries. The SADC includes a diverse set of countries including the island nations of Seychelles, Mauritius, and Madagascar. Efforts should be made to prioritize important aquatic systems such as fragile habitats, conservation areas, waterways, areas designated for human use, and ecosystem services. In addition, robust sampling campaigns should be conducted to better understand chemical exposure in these key areas.

There is currently a paucity of knowledge on the occurrence of emerging contaminants in wastewater, surface water, and particularly groundwater in the SADC. The lack of groundwater data is especially concerning given that rapid population growth, climate change, and socio‐economic growth are likely to increase dependence on groundwater in the future (Barbieri et al., 2019). Such data are critical for understanding the impact of emerging contaminants on groundwater quality. Today, many people in Southern Africa still rely on pit latrines, dug‐out wells, and open defecation. These activities directly affect groundwater quality and (depending on proximity) may act as a source of diffuse pollution to surface water. Because groundwater remediation remains a complex and potentially expensive endeavour, investments should be made in enforcing environmental regulations and improving education and sanitation technologies to reduce potential exposure.

Although data on emerging contaminants from the region are sparse, with only 226 unique compounds monitored, several unique classes of emerging contaminants are completely absent. Interestingly, no investigations have been carried out on illicit drugs and their residues in the environmental waters of Southern Africa, even though they have been detected in untreated wastewater in South Africa (Archer et al., 2018), in groundwater (Jurado et al., 2012), and in drinking water (How & El‐din, 2021) in other parts of the world. Illicit drugs have also been classified as a class of emerging contaminants, with the potential to cause unwanted effects at low concentrations (Reid et al., 2014). There is growing concern that many of their degradants, metabolites, and treatment byproducts may still retain bioactivity while in the environment (González‐Mariño et al., 2013). The analysis of drugs in wastewater can also be very helpful in providing a vast amount of knowledge helpful for monitoring usage in a given population or area. This technique, termed wastewater‐based epidemiology (WBE), involves measuring the concentrations of compounds of interest in influent waters of treatment facilities, and relating these concentrations to local consumption rates (Reid et al., 2014). Although WBE approaches are gaining popularity in different parts of the world, only one such study (Archer et al., 2018) has been conducted in Southern Africa. The WBE technique may be especially informative in the SADC, where national usage data on chemical classes such as pharmaceuticals do not exist for most countries—as evidenced by the present study.

To efficiently monitor levels of emerging contaminants and pollution, it is important to develop prioritization lists of emerging contaminants in environmental waters (Guo et al., 2016). The Water Framework Directive is an example of such a prioritization list that identifies substances of environmental concern according to set environmental quality standards for environmental waters within the European Union (European Commission, 2013). However, no such methods have been applied by the SADC and thus, there is an urgent need for the elucidation of such priority models that will focus both research and the development of governmental policies on environmental water contaminants based specifically on the unique ecological and socio‐economic activities of Southern Africa. Longitudinal studies of regular monitoring should be established for priority surface waters to gain a better understanding of pollution dynamics, but also to evaluate future trends and the potential impacts of mitigation programs.

Similarly, to improve the reproducibility and overall quality of results obtained, it is important to develop standardized protocols of environmental water sampling techniques and storage methods in the SADC. Although this may be difficult given particular and variable conditions during data collection, and because specialized equipment may be limited, the use of various controls can help validate sampling robustness. For example, field spikes can be used as positive controls, whereby known concentrations of analytical standards are combined with contaminant‐free water at the same time that samples are collected; thus all handling and storage processes are performed as samples. In this way, the stability of analytes can be monitored after sample collection and could help overcome issues related to sample storage. In addition, standardizing the use of amber glass vials over clear glass bottles or plastic jars to minimize contamination, standardizing filtration of samples before storage, and storage of samples at –20 °C will help to increase accuracy of the data (González‐Mariño et al., 2010). In many of the studies under review, only vague details such as the name of the river and the date of sampling were reported. An ideal sampling protocol would improve this situation by only allowing reports with clear accounts of parameters such as weather, exact location of sampling (GPS coordinates, depth, and distance from the bank), time, and date.

Globally, there remains a general lack of knowledge regarding the transformation of emerging contaminants in the environment through processes such as biotransformation, photolysis, sorption, desorption, dispersion, or volatilization (Mackie et al., 2017). These processes not only impact the exposure and occurrence of the parent compound, but also may result in the transformation of these compounds into a suite of degradants and metabolites generally referred to as transformation products (Boix et al., 2014). However, due to the abundance of possibilities of transformation pathways (resulting in an overwhelming number of potential transformation products), it remains a challenge to accurately assess their environmental impacts because there is a general lack of data on exposure and effects of transformation products. Determination of the environmental fate of representative chemical groups will aid in the development of suitable analytical methods and also provide guidance for the toxicological assays. Furthermore, the adaptation of high‐resolution MS in the environmental sciences allows for nontargeted or suspect screening of the entire extractable and ionizable chemical exposome (Pereira et al., 2021). Nontargeted analysis is typically a semiquantitative approach, but could help to inform prioritization efforts in the regions that lack national chemical inventories and usage or consumption data.

In 2017, the United Nations Environment Assembly committed to a “pollution‐free planet” by 2030 (UNEP, 2017). Good progress is being made in the SADC through multilateral environmental agreements such as the Basel, Stockholm, Rotterdam, Bamako, and Minamata Conventions, the Strategic Approach to International Chemicals Management, and initiatives such as that of the Africa Institute and the Institute of Wastewater Management. However, more needs to be done. According to the Millennium Assessment Report of United Nations (2012), only 15% of the SADC countries are on track to meet the SDGs target on sanitation (United Nations, 2012). If Southern Africa is to meet this target, urgent action is needed to develop policies, create innovative programs, and implement best practices to clean up rivers, lakes, and coastlines to reduce pollution from all sources. Governments and health agencies need to pay more attention to environmental pollution, and regulatory authorities in sub‐Saharan Africa need to be more stringent in enforcing existing laws. Scientists and residents need to become better informed about the extent of contamination to trigger precautionary protective behavior. Politicians must prioritize installing and maintaining infrastructure capable of treating pollution—commensurate with the rate of urban growth. Governments can consider approaches taken in the European Union, China, Philippines, and the United States to ban chemicals in products to reduce pollution (e.g., phosphates in detergents and recent actions against nonessential use of PFAS), as well as implement fiscal incentives, tighten up standards, scale up analytical capabilities and equipment, build the legitimacy of strong institutions, and incentivize sustainable lifestyle choices (Brannigan, 2019). Taxpayers' money can be used to buy up land with high nutrient loads to regenerate forests and encourage land managers to employ innovative pollution control and reduction practices (Walker, 2019), as in the case of Lake Tuapo in New Zealand. Finally, developed nations outside the SADC have a critical role in stopping the long‐standing practice of exporting toxic waste to Southern Africa.

## CONCLUSIONS

The present study provides a critical overview on the current state of knowledge on the exposure of chemical contaminants in environmental waters in the SADC, with a focus on emerging contaminants. Our study has assessed the spatial distribution of monitoring studies, the chemical classes, and the analytical techniques used to analyze pollutants. A total of 59 investigations were used to develop the database for analysis. The highest number of articles came from South Africa (*n* = 36) > Botswana (*n* = 6) > Zimbabwe (*n* = 6) > Malawi (*n* = 3) > Mozambique (*n* = 3) > Zambia (*n* = 2) > Angola (*n* = 1) > Madagascar (*n* = 1) > Tanzania (*n* = 1). No publications were found from Comoros, Democratic Republic of Congo, eSwatini, Lesotho, Mauritius, Namibia, or the Seychelles. Of these studies, 47% (28 articles) included emerging contaminants, of which only 2 studies were conducted outside of South Africa. The emerging contaminant with the highest average concentration was the antiretroviral drug ritonavir (64.52 µg/L), whereas the compound detected the most times was ibuprofen (17 times). Groundwater systems were least investigated (13 studies) despite being the primary water source for humans and wildlife in the area. On the other hand, surface water (48 studies) and wastewater (15 studies) were studied more extensively. High emerging contaminant concentrations in surface water indicates low removal efficiencies in WWTPs and the presence of secondary sources of pollution.

As the SADC balances ambitious goals for economic growth with commitments to the SDGs, it is important that chemical pollution not be ignored. Regional and international collaboration may be key to urgently address the significant gaps in the assessment of chemical exposure, especially in SADC countries outside of South Africa. Development of local capacity to assess emerging contaminant pollution is ultimately required to ensure the safety and security of water resources into the future.

## Data Availability

The database is openly accessible (https://pure.york.ac.uk/portal/files/73799193/Review_Database.xlsx). Other data, associated metadata, and calculation tools are available from the corresponding author (brett.sallach@york.ac.uk).
